# Socioeconomic Patterns in Budget Share Allocations of Regulated Foods and Beverages in Chile: A Longitudinal Analysis

**DOI:** 10.3390/nu15030679

**Published:** 2023-01-29

**Authors:** Guillermo Paraje, Daniela Montes de Oca, Camila Corvalán, Barry Popkin

**Affiliations:** 1 School of Business, Universidad Adolfo Ibáñez, Avenida Diag Las Torres 2640, Santiago 7941169, Chile; 2 Instituto de Nutriciόn y Tecnología de los Alimentos (INTA), Universidad de Chile, Santiago 7830490, Chile; 3 Department of Nutrition, University of North Carolina at Chapel Hill, 123 W Franklin St, Suite 210, CB 8120, Chapel Hill, NC 27516, USA

**Keywords:** budget share allocation, food labeling, food regulation, Chile

## Abstract

Chile has enacted stringent legislation regulating food and beverage labeling and advertising. This study assesses the changes in the average relative allocations of food and beverage budgets for regulated versus not regulated products in households of different socioeconomic and demographic backgrounds. A household fixed effect before–after model is estimated and the marginal effects in the changes of levels and trends in budget shares and purchased volumes after the implementation of the regulations are examined. The results highlight how impactful food policies can shift consumption toward healthier products.

## 1. Introduction

In 2016 the Chilean government enacted Law 20,606 to curb high levels of people overweight or with obesity. The regulations in the law include front-of-package octagonal warning labels on manufactured foods and beverages high in added sugars, sodium, saturated fats, and calories if they surpassed predefined cutoffs [1]. These “high-in” cutoffs became increasingly stringent over a 4-year period of enacting the law in 3 phases: phase 1 from 27 June 2016; phase 2 from 27 June 2018; and phase 3 from 27 June 2019. In addition, the law banned sales of products with any front-of-package label in schools and very comprehensively banned marketing these products to children (using cartoons, toys, etc.). These regulations aimed to provide easy-to-understand nutritional information for food and beverage products and protect children under the age of 14 from labeled products’ advertising and availability in schools.

A growing body of literature has shown that the population, especially mothers of young children [2,3], received these measures well and that the regulations produced significant decreases in the volume of certain food categories purchased. For instance, the volume of sugar-sweetened beverages (SSBs) purchased decreased by approximately 25% [4]. Unhealthy food purchases declined significantly overall [5], and many food categories, such as breakfast cereals, decreased strongly [6,7]. Product reformulations were also extensive and significant, as the proportion of products with any high-in label decreased from 51% to 44% [8]. These findings are in line with what other countries have experienced. A recent meta-analysis found that food labeling is effective in reducing consumers’ intakes of energy, total fat, and other unhealthy options while increasing industry reformulations to decrease sodium and trans fat content [9].

Though these regulations were effective in reducing the intake of regulated ingredients, they did not affect labor market outcomes. Studies have found that after the implementation, neither aggregate employment nor real wages in the affected sectors changed when compared to their evolution in nonaffected sectors [10,11]. Other variables, such as capital expenditures and physical production, in the affected sectors did not significantly change in levels or in trends after the implementation of the law when compared to nonaffected sectors [12].

However, the evidence does not reveal how households changed their budget allocations in monetary terms after the implementation of Law 20606. The law’s impact on relative budget allocations between labeled foods and beverages and non-labeled products is unknown. In principle, a decrease in the physical quantities of labeled products purchased, as found elsewhere [5], could have been compensated by an increase in the relative prices of such items, resulting in an increase in those items’ household budget shares. In such a case, it could be said that the implementation of Law 20606 had a negative financial impact on households and that the impact could be relatively higher on poorer households. However, preliminary results show that the regulations did not change relative prices of labeled versus unlabeled products for poorer households [13].

If lower socioeconomic households do not change their budget allocations between labeled and unlabeled products, while higher socioeconomic households do, it may imply that the former do not have ready unlabeled alternatives to do so or that they are relatively more expensive. Studying changes in budget allocations may show whether regulations have differential impacts and provide more evidence of regulations’ outcome inequalities.

This manuscript assesses the changes in the average relative food and beverage budget allocations for labeled versus non-labeled products in households of different socioeconomic and demographic backgrounds during the first stage of the implementation of Law 20606 between July 2016 and December 2017. In addition, and as a byproduct, it analyzes the changes in the average volume of foods and beverages households of different socioeconomic characteristics purchased.

This study is the first to investigate the relationship between Law 20606 and household budget allocations for foods and beverages. We are not aware of studies of this relationship in other countries. Our working hypotheses are that the enactment of the regulations were associated with a decrease in the volume of labeled products purchased and a decrease in the budget shares allocated to labeled products. We have no evidence of price changes associated with the regulations; consequently, changes in volumes would translate into changes in budget shares. In addition, we expect no significant differences in these changes by socioeconomic status (SES) levels as we find no evidence of SES differences in the availability of healthy products in Chile.

## 2. Materials and Methods

### 2.1. Data

We used longitudinal information that Kantar Worldpanel collected on food and beverage purchases at the household level from 1 January 2014 to 31 December 2017. This data set has been used elsewhere to evaluate different aspects of Law 20606 [4,5,8]. Kantar Worldpanel also collected household SES data, including size, ages and genders of members, education level of the head of the household, household assets, and access to services for 2573 households. Kantar Worldpanel presents households in the panel for a median time of 18 months and on average includes 1936 households per month. The sample is representative of urban areas with more than 20,000 habitants, about 74% of the urban population.

Kantar Worldpanel bases household SES on access to a list of goods and services and the education level of the head of the household. The ABC1 group is the highest SES level, representing about 15% of the sample; the DE group is the lowest, representing about 32% of the sample; and the C2C3 group is the middle, representing about 53% of the sample. In addition, Kantar Worldpanel provides a life cycle variable based on the household’s demographic structure, categorized as (a) households without children; (b) couples with the youngest child up to 5 years old; (c) couples with the youngest child 6 to 12 years old; (d) couples with the youngest child 13 to 17 years old; (e) couples with the youngest child 18 to 29 years old; and (f) monoparental households with children.

Kantar Worldpanel interviewers visited households weekly to collect data on food and beverage purchases using a handheld barcode scanner. The data include volume in milliliters (mL) or weight in grams (g), price per unit, brand (with a unique barcode), package size, and date of purchase. Our analysis period registered 92,962 household-month observations and 163 purchased product categories. As described elsewhere [5,14], each product’s nutrition facts panel was linked to household purchases using barcode, brand, and product description, and a team of trained nutritionists reviewed each product to determine if it was high in sugars, sodium, or saturated fats or exceeded phase 1′s calorie threshold. In this way, every product a household purchased before phase 1 of the implementation of Law 20606, January 2014–June 2016, was categorized as high or not high in regulated ingredients based on the criteria on 27 June 2016, when implementation began.

### 2.2. Statistical Methods

The outcome variable we considered is the monthly budget share of labeled products. For the analyses we defined several groups of products: (a) unlabeled (no label); (b) labeled (with at least 1 label); (c) high in calories (with at least that label); (d) high in sodium (with at least that label); (e) high in sugars (with at least that label); and (f) high in saturated fats (with at least that label). Groups are not mutually exclusive (apart from unlabeled versus labeled products, which are mutually exclusive) and products are assigned to all the groups that apply to them. For example, a product with 2 labels is assigned to the labeled group and the 2 groups representing its labels.

We defined the budget share of labeled products as the proportion of the average household food and beverage budget allocated to labeled products. That variable is constructed as the ratio between the monthly expenditures on labeled products and the monthly expenditure on the complete food and beverage basket:

(1) $$B_{t}^{H}=\frac{\sum_{i=1}^{163}P_{i,t}^{H}Q_{i,t}^{H}}{M_{t}}$$

where $B_{t}^{H}$ represents the budget share of labeled products in month *t*; $P_{i,t}^{H}$ is the price in month *t* of the labeled product *i* (not all of our 163 purchased product categories were labeled products); $Q_{i,t}^{H}$ is the quantity purchased in month *t* of the labeled product *i*; and $M_{t}$ is the monthly total budget allocated to all foods and beverages consumed at home.

Exploiting the panel data, we estimated a household fixed effect before–after model taking the following form:

(2) $$B_{jt}=\alpha +\beta_{1}Law_{t}+\beta_{2}T_{t}+\beta_{3}Law_{t}*T_{t}+\overset{2}{\underset{i=1}{\sum }}\gamma_{i}SES_{ijt}+\overset{2}{\underset{k=1}{\sum }}\gamma_{k}SES_{kjt}*Law_{t}+\;\overset{2}{\underset{l=1}{\sum }}\gamma_{l}SES_{ljt}*T_{t}+\overset{2}{\underset{m=1}{\sum }}\gamma_{m}SES_{mjt}*Law_{t}*T_{t}+\overset{5}{\underset{n=1}{\sum }}\phi_{n}Cycle_{njt}+\;\overset{5}{\underset{o=1}{\sum }}\phi_{o}Cycle_{ojt}*Law_{t}+\overset{5}{\underset{p=1}{\sum }}\phi_{p}Cycle_{pjt}*T_{t}+\overset{5}{\underset{q=1}{\sum }}\phi_{q}Cycle_{qjt}*Law_{t}*T_{t}+\;\overset{3}{\underset{s=1}{\sum }}\delta_{s}HH_{sjt}+\overset{11}{\underset{u=1}{\sum }}\rho_{u}D_{ut}+c_{j}+u_{jt}$$

where $B_{jt}$ is the budget share of labeled products for household *j* in month *t* as defined in (1); $Law_{t}$ is a dichotomous variable with a value of 1 from July 2016 onward and 0 otherwise; $T_{t}$ is a monthly trend; $SES_{jt}$ is a set of dichotomous variables recording the SES level of household *j* at month *t* (ABC1 is the reference category); $Cycle_{jt}$ is a set of dichotomous variables recording the life cycle variable of household *j* at month *t* (household without children is the reference category); $HH_{sjt}$ is a set of household variables that includes the size of the household, the age of the household head, and the proportion of household members overweight or obese; and $D_{ut}$ is a set of dichotomous variables for calendar months. The term $c_{j}$ is a household fixed effect to control for households’ unobserved characteristics.

We estimated a similar model for the purchased monthly total volume of labeled solid foods (g) and beverages (mL). The dependent variable is simply the sum of the volume (g or mL) of labeled products. This breakdown is important as beverage volumes are much greater than those of food and beverage volume shifts can hide food volume changes in the total volume analysis. The results of the models for volume purchases are in the Supplemental Online Material.

We used the “xtreg” command in Stata 17 to estimate models. We used the “margins” command in Stata 17 to estimate marginal effects in the changes of levels after the implementation of Law 20606 and trends after that moment compared to previous existing trends. We estimated such margins for the SES and life cycle variables. To compare marginal effects across SES levels and/or across life cycle categories, we performed standard *t*-tests for equality of marginal effects (*m*) using the delta method [15,16,17]:

$$Z=\frac{m_{1}-m_{2}}{\sqrt{\left(se_{m1}^{2}+se_{m2}^{2}\right)}}$$

## 3. Results

Table 1 shows the characteristics for the total sample and by SES level. The average household size of the sample was 4.12 members. The ABC1 group, the highest SES level, averaged 3.82 members and the DE group, the lowest SES level, averaged 4.36 members. More than 40% of the households were in Santiago, 11% were in Valparaíso, 12% were in the central south region, 11% were in the Biobío region, 12% were in the south, and 11% were in the north. Households with no children were 21% of the total sample and were more prevalent in the ABC1 group, whereas households with at least one child younger than five were 27% of the sample and were more prevalent in the DE group.

**Figure 1:**
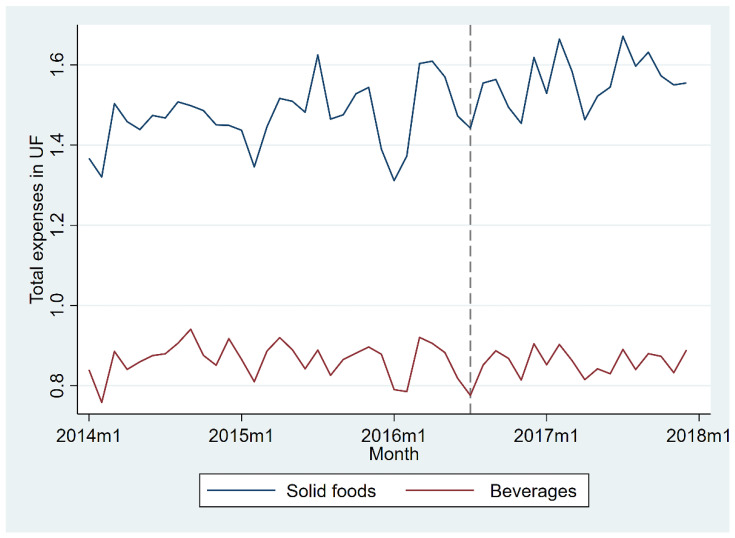
Total real expenditures allocated to foods and beverages.

The budget share allocated to foods and beverages was constant across this period in real terms. Figure 1 displays the evolution of the average real budget share allocated to foods and beverages, regulated or not, between January 2014 and December 2017. All prices are converted from current Chilean pesos to Unidades de Fomento (UF), a constant currency unit adjusted daily for inflation used in Chile to update values like wages, mortgages, and loans. July 2016 was the first full month of phase one of the regulations. Our before–after analysis showed no change in either levels or trends for the real expenditures on foods and beverages (not shown but available from the authors). This may indicate that, keeping expenditures constant, any changes are reallocations between labeled and non-labeled products.

Table 2 shows the average budget share allocated to labeled products preintervention, January 2014–June 2016, and postintervention, July 2016–December 2017, for the total sample and by SES level. In the preintervention period, more than 57% of an average household’s food and beverage budget was allocated to products that would not have had any labeling and it increased to 67% after the intervention, a statistically significant difference. Similarly, the food and beverage budget allocated to any labeled products decreased after the intervention, driven by products with labels for saturated fats, sodium, and sugars. We observed minor increases in products with labels for calories. All SES levels exhibited this pattern though with different intensities.

Table 3 shows the marginal effects for the total sample. Regarding Equation (2), the table shows the derivative of the dependent variable over the variable law. Complete results for Equation (2) are in Supplemental Material Table S1. The change in the level of the budget share allocated to labeled products decreased 9.6 percentage points (*p* < 0.01) right after the intervention (July 2016). The decrease was 1.2 percentage points (*p* < 0.01) for products with at least the label for calories, 3.0 percentage points (*p* < 0.01) for those with at least the label for saturated fats, 5.3 percentage points (*p* < 0.01) for products with at least the label for sodium, and 7.2 percentage points (*p* < 0.01) for products with at least the label for sugars. Results are in Supplemental Material Table S2.

After the implementation of the front-of-package label regulations, the budget share allocation trends changed significantly. Preintervention, the positive trend in spending more on foods and beverages that would have had warning labels was equal to 0.0004 (*p* < 0.0100) per month. Postintervention, the trend became negative, equal to −0.0005 (*p* < 0.0100). Products at least labeled for calories had a preintervention trend of growth in budget share allocations but postintervention experienced a significant decrease. The trend in products at least labeled for sugars was already decreasing preintervention and decreased further postintervention. Results are in Supplemental Material Table S2. Products labeled for saturated fats saw an increase in the positive postintervention trend relative to the preintervention one. Those labeled for sodium showed no change between pre- and postintervention trends.

Table 4 displays the marginal effects of changes in levels and trends over SES groups. In all cases, budget shares of labeled products fell with the intervention from 9.2 percentage points for the C2C3 group to 10.1 percentage points for the ABC1 group. However, differences among groups are not statistically significant. Trends in budget share allocations for labeled products did not change with the intervention in the ABC1 group, which remained flat. For the other 2 groups, trends in budget share allocations for labeled products decreased similarly (*p* < 0.01 in both cases) with the intervention.

Regarding products labeled for specific nutrients, in the ABC1 group the only statistically significant trend change was for saturated fats (*p* < 0.05), which moderately increased after the intervention. Results are in Supplemental Material Table S3. In the C2C3 group, only products labeled for calories showed a significant trend change (*p* < 0.01), which decreased with the intervention. The DE group showed decreases in the trends for calories (*p* < 0.10) and sugars (*p* < 0.05) and an increase in the trend for saturated fats (*p* < 0.10).

Table 5 shows the marginal effects of the implementation of the law over the life cycle variable. Households with at least one child younger than five had the largest decrease in the budget share allocated to labeled products. Right after the intervention, the share fell 11.9 percentage points (*p* < 0.01). Households with children 6–12 years old and 13–17 years old also decreased their budget shares allocated to labeled products by more than 10.0 percentage points. In trend changes, monoparental households with children showed the largest trend reductions in budget shares allocated to labeled products.

When we analyzed per label, the largest changes were in high-in sugar products, and the smallest were in high-in calories products. That was true for all SES levels. Results are in Supplemental Material Tables S2 and S3. 

Results for volume are in Supplemental Material Tables S4–S10. Labeled products saw significant decreases in volume purchases, while unlabeled products registered the opposite. Monthly purchases of labeled solid foods decreased by 1.5 kg after the intervention, while monthly purchases of labeled beverages decreased by 3.6 L. In addition, after the intervention the trend in monthly purchases of labeled solid foods and beverages decreased significantly (*p* < 0.01). For instance, the preintervention trend in volume purchases of labeled beverages implied a monthly decrease of 50 mL and that trend accelerated to 93 mL with the intervention. Marginal effects of volume purchases also show changes across SES groups, all of which had statistically significant decreases in volume purchases after the implementation of regulations. Statistical comparisons among groups show that decreases in volume purchases were higher for the ABC1 group when compared to the DE group in all cases. Finally, all family types show decreases in volume purchases of labeled products, though the highest drops were in households with children 6–12 and 13–17 years old.

## 4. Discussion

Using methods different from those of previous studies, we confirmed in this study that the enactment of Law 20606 significantly impacted household purchases of foods and beverages with warning labels [5]. Using a household-fixed effects panel data model to estimate before–after marginal effects of the regulations implemented at the end of June 2016, we found that Chileans immediately decreased both the volume of labeled products purchased and the proportion of households’ budgets allocated to them. The reductions were statistically significant and generally a reduction in the trends of monthly purchases followed. We found no statistical differences in the magnitude of the reduction in budget shares allocated to labeled products across SES groups. Budget shares changed in a context of constant real household expenditures, which in practical terms means that households decreased real expenditures on regulated foods and beverages.

Results should be considered in the context of changes in firms’ marketing strategies and product reformulations [3,8]. In response to the regulations, firms may have reduced and changed marketing campaigns, reformulated products to avoid the regulations, or changed prices of both regulated and nonregulated products. For instance, in the case of marketing, evidence shows that after the regulations, children’s exposure to high-in food media advertisements decreased by 44% for preschoolers and 58% for adolescents with significant decreases in child-directed appeals, such as cartoons [18]. TV ads for high-in products decreased significantly. Before the regulations, 42% of food advertisements on TV were for high-in products compared with 15% after the regulations [19]. The prevalence of child-directed appeals on packages of certain products, such as breakfast cereals, decreased significantly after the implementation of the law [20].

Additionally, the product reformulations that were extensive at least during phase one of the law’s implementation [8] could have impacted costs and prices, though a recent study conducted with the same database we used found that prices of reformulated products did not change vis-à-vis non reformulated products [13]. Constant relative prices between regulated and nonregulated foods and beverages suggest that decreases in the volumes purchased account for almost all of the regulations’ effects on household budget shares.

Though decreases in purchased volumes were significant, they do not seem to be enough to reverse the high levels of people overweight and obesity in Chile, which is currently 75% of the population [21]. Law 20606 achieved many of its goals, including educating the population about regulated ingredients, prompting reformulation of products, and restricting child-oriented marketing, among others. However, further decreasing consumption of products high in regulated ingredients may require measures that affect relative prices, such as taxes. Currently, SSBs are the only food or beverage taxed and have a relatively low excise taxes that have minor effects on consumers’ purchases [22].

A frequent concern about imposing taxes on food and beverage products is the negative financial impact (i.e., regressivity) that increased prices may have on lower income households [23]. A counterargument is that overweight/obesity is more prevalent among lower SES households and reduced consumption of unhealthy products would lower present and future expenditures on health care. Chile has a clear negative gradient between years of education and overweight/obesity [21]. Studies on the distributive effects of SSB taxation have found that taxes are progressive, as lower income households’ positive effect of lower health care expenditures outweigh any financial burden due to higher taxes [24,25,26].

Our results show no large SES differences in how households reacted to food regulations. A recent umbrella review found, apart from taxation that reduces SES inequalities, no compelling evidence of food regulation effects on inequalities [27]. Our results show that in terms of budget shares and volumes, the policies adopted in Chile had a uniform socioeconomic effect despite arguments that less-educated individuals and families would not change their habits in response to regulations.

Although we found no changes in budget allocation patterns based on SES after the intervention, this does not mean that other kinds of regulations, such as taxes on unhealthy foods and beverages, will not impact those patterns. Future research should investigate how these policies could affect household choices.

This analysis has some limitations. First, Law 20606 is a package of regulations, including labeling and advertising bans, and the individual effects of each regulation on households’ expenditures cannot be separated. Second, the basket in the Kantar Worldpanel database corresponds to one-third of the total food and beverage basket, mostly from supermarket purchases, that the Institute of National Statistics considers because the Kantar Worldpanel data set does not include information on purchases of bulk products, such as fruits, vegetables, meats, and breads. We have no evidence that the regulations changed the proportions of foods and beverages bought from supermarkets, smaller stores, or other sources. As such, one can assume that the regulations did not alter the patterns of purchases and that the changes reported here effectively reflect changes at the population level. Third, there has been a significant time gap with the situation described here and the current situation. In that period, there was not only COVID but also a political shock due to social unrest in Chile (from October 2019 to the appearance of COVID in March 2020). We are not claiming that changes in budget allocation produced by the initial implementation of the Law remained until today. We acknowledge that that is unlikely and that many other factors may have affected such allocation. However, such factors (COVID, social unrest, etc.) are not related to the implementation of the Law (exogenous factors) and, as such, should not be considered in an enquire about the effects of the Law.

## 5. Conclusions

The enactment of Law 20606 has been called “the world’s most ambitious attempt to remake a country’s food culture” [28,29]. It decreased volume purchases of products with warning labels and incentivized product reformulations while having no discernible effects on aggregate levels of employment or real wages. This article shows that the enactment of the law was associated with changes in households’ budget allocations as they reduced the shares spent on labeled products. The reduction was similar across households’ SES levels and demographic compositions.

## Figures and Tables

**Table 1:** Characteristics and distribution of the total sample and by SES level.

		ABC1	C2C3	DE	Average Number of Total Households (Monthly)
Monthly average number of households		292.91	1014.45	626.08	1933.45
		(8.67)	(23.13)	(16.53)	(31.71)
Distribution		0.15	0.53	0.32	
		(0.36)	(0.50)	(0.47)	
Region	North	0.10	0.12	0.11	0.11
		(0.30)	(0.31)	(0.31)	(0.32)
	Central south	0.90	0.88	0.89	0.88
		(0.30)	(0.33)	(0.31)	(0.32)
Female head of household		0.39	0.42	0.46	0.43
		(0.49)	(0.49)	(0.60)	(0.50)
Head of household’s age	Mean	58.29	57.38	53.46	56.25
		(14.47)	(15.26)	(15.42)	(15.32)
Household size	Mean	3.85	4.06	4.36	4.13
		(1.47)	(1.67)	(1.72)	(1.68)
Monthly average of households per life cycle					
No children		0.27	0.23	0.15	0.21
		(0.44)	(0.42)	(0.36)	(0.41)
Couple with children < 5 years		0.17	0.25	0.36	0.27
		(0.37)	(0.43)	(0.48)	(0.45)
Couple with children 6–12 years		0.17	0.18	0.16	0.17
		(0.38)	(0.38)	(0.37)	(0.38)
Couple with children 13–17 years		0.13	0.10	0.08	0.09
		(0.34)	(0.29)	(0.26)	(0.29)
Couple with children 18–29 years		0.19	0.13	0.08	0.12
		(0.39)	(0.34)	(0.27)	(0.33)
Monoparental with children		0.08	0.12	0.17	0.13
		(0.27)	(0.32)	(0.38)	(0.34)

Standard deviations in parentheses.

**Table 2:** Average monthly budget share allocated to labeled products by period and differences of means, total sample, and by SES level.

Total Sample						
	No Label	Any Label	Calories	Saturated Fats	Sodium	Sugars
Preintervention average	0.5740	0.4260	0.1390	0.1330	0.0860	0.2670
	(0.0140)	(0.0140)	(0.1100)	(0.0040)	(0.0040)	(0.0150)
Postintervention average	0.6670	0.3330	0.1430	0.1160	0.0300	0.1900
	(0.0120)	(1220)	(0.0050)	(0.0030)	(0.0020)	(0.0110)
Difference of means	0.0890	−0.0930	0.0040	−0.0170	−0.0550	−0.0770
Standard error	0.0040	0.0040	0.0030	0.0010	0.0010	0.0040
**ABC1 households**						
	No Label	Any Label	Calories	Saturated Fats	Sodium	Sugars
Preintervention average	0.5999	0.4001	0.1533	0.1441	0.0782	0.2299
	(0.1333)	(0.1333)	(0.0796)	(0.0804)	(0.0626)	(0.1235)
Postintervention average	0.6998	0.3001	0.1583	0.1249	0.0265	0.1379
	(0.1294)	(0.1294)	(0.0887)	(0.0801)	(0.0372)	(0.0990)
Difference of means	0.0999	−0.0999	0.0050	−0.0192	−0.0518	−0.0921
Standard error	0.0023	0.0023	0.0015	0.0014	0.0010	0.0020
**C2C3 households**						
	No Label	Any Label	Calories	Saturated Fats	Sodium	Sugars
Preintervention average	0.5772	0.4228	0.1466	0.1376	0.0821	0.2608
	(0.1442)	(0.1442)	(0.0836)	(0.0813)	(0.0662)	(0.1371)
Postintervention average	0.6736	0.3264	0.1488	0.1177	0.0277	0.1808
	(0.1392)	(0.1392)	(0.0921)	(0.0768)	(0.0393)	(0.1201)
Difference of means	0.0964	−0.0964	0.0022	−0.0199	−0.0544	−0.0800
Standard error	0.0013	0.0013	0.0008	0.0007	0.0005	0.0012
**DE households**						
	No Label	Any Label	Calories	Saturated Fats	Sodium	Sugars
Preintervention average	0.5686	0.4314	0.1290	0.1295	0.0905	0.2753
	(0.1597)	(0.1597)	(0.0817)	(0.0822)	(0.0711)	(0.1549)
Postintervention average	0.6553	0.3447	0.1356	0.1161	0.0331	0.2052
	(0.1525)	(0.1525)	(0.0927)	(0.0803)	(0.0415)	(0.1405)
Difference of means	0.0866	−0.0866	0.0065	−0.0134	−0.0574	−0.0701
Standard error	0.0019	0.0019	0.0010	0.0010	0.0007	0.0018

Standard deviations in parentheses.

**Table 3:** Marginal effect on budget shares and trends before and after the intervention.

	Change in Level Postintervention	Trend		
		Preintervention Trend	Postintervention Trend	Difference between Post- and Preintervention Trends
Any label	−0.0962 ***	0.0004 ***	−0.0005 ***	−0.0009 ***
	(0.0026)	(0.0001)	(0.0002)	(0.0002)

*** *p* < 0.01. Standard errors in parentheses.

**Table 4:** Marginal effects on the budget shares and trends before and after the intervention by SES level.

	Change in Level ABC1	Change in Level C2C3	Change in Level DE
Any label			
	−0.1013 ***	−0.0927 ***	−0.1000 ***
	(0.0060)	(0.0034)	(0.0046)
Difference	ABC1 vs. C2C3	C2C3 vs. DE	ABC1 vs. DE
	−0.0086	0.0072	−0.0014
	(0.0067)	(0.0056)	(0.0074)
	ABC1	C2C3	DE
Preintervention trend			
	−0.0001	0.0002 **	0.0009 ***
	(0.0002)	(0.0001)	(0.0001)
Postintervention trend			
	−0.0001	−0.0008 ***	−0.0002
	(0.0004)	(0.0002)	(0.0003)
Difference between post- and preintervention			
	−0.0001	−0.0011 ***	−0.0011 ***
	(0.0004)	(0.0002)	(0.0003)

** *p* < 0.05, *** *p* < 0.01. Standard errors in parentheses.

**Table 5:** Marginal effects on budget shares and trends before and after the intervention by life cycle.

	Households without Children	Couple with Children < 5 Years Old	Couple with Children 6–12 Years Old	Couple with Children 13–17 Years Old	Couple with Children 18–29 Years Old	Monoparental with Children
Change in level	−0.0861 ***	−0.1190 ***	−0.1052 ***	−0.1079 ***	−0.0866 ***	−0.0949 ***
	(0.0041)	(0.0181)	(0.0057)	(0.0080)	(0.0053)	(0.0065)
Preintervention trend	0.0004 ***	−0.0001	0.0004 ***	0.0001	0.0002	0.0009 ***
	(0.0001)	(0.0005)	(0.0002)	(0.0002)	(0.0002)	(0.0002)
Postintervention trend	−0.0003	−0.0001	−0.0005	−0.0002	−0.0011 ***	−0.0005
	(0.0003)	(0.0009)	(0.0003)	(0.0004)	(0.0003)	(0.0004)
Difference between pre- and postintervention	−0.0007 **	0.0000	−0.0009 **	−0.0003	−0.0013 ***	−0.0015 ***
	(0.0003)	(0.0010)	(0.0004)	(0.0005)	(0.0004)	(0.0005)

** *p* < 0.05, *** *p* < 0.01. Standard errors are in parentheses.

## Data Availability

The data used in the study are licensed and not publicly available.

## Supplementary Materials

The following supporting information can be downloaded at: https://www.mdpi.com/article/10.3390/nu15030679/s1, Table S1: Complete results for the estimation of Equation (2) for budget share; Table S2: Marginal effect on the budget shares and trend before and after the intervention by type of labels; Table S3: Marginal effect on the budget shares and trend before and after the intervention by type of labels and SES level; Table S4: Average monthly volume purchased of high in- products by period and by SES level; Table S5: Marginal effect of the intervention on the volume purchased of labelled products and trends before and after the intervention; Table S6: Marginal effect of the intervention on the volume purchased and trend before and after the intervention by label and by SES level; Table S7: Marginal effect of the intervention on the volume purchased and trend before and after the intervention by "life cycle"; Table S8: Marginal effect of the intervention on the monthly volume purchased in labelled products and trends before and after the intervention by type of label; Table S9: Marginal effect of the intervention on the volume purchased and trend before and after the intervention by type of label and by SES level; Table S10: Marginal effect of the intervention on the volume purchased and trend before and after the intervention by label and by life cycle.

## References

[1] Gobierno de Chile (2016). Ley 20606: Sobre Composición Nutricional de los Alimentos y su Publicidad.

[2] Subsecretaría de Salud Pública (2017). Informe de Evaluación de la Implementación de la Ley Sobre Composición Nutricional de los Alimentos y su Publicidad.

[3] Correa T., Fierro C., Reyes M., Carpentier F.R.D., Taillie L.S., Corvalan C. (2019). Responses to the Chilean law of food labeling and advertising: Exploring knowledge, perceptions and behaviors of mothers of young children. Int. J. Behav. Nutr. Phys. Act..

[4] Taillie L.S., Reyes M., Colchero M.A., Popkin B., Corvalán C. (2020). An evaluation of Chile’s Law of Food Labeling and Advertising on sugar-sweetened beverage purchases from 2015 to 2017: A before-and-after study. PLoS Med..

[5] Taillie L.S., Bercholz M., Popkin B., Reyes M., Colchero M.A., Corvalán C. (2021). Changes in food purchases after the Chilean policies on food labelling, marketing, and sales in schools: A before and after study. Lancet Planet. Health.

[6] Araya S., Elberg A., Noton C., Schwartz D. (2022). Identifying Food Labeling Effects on Consumer Behavior. Mark. Sci..

[7] Vatter B., Barahona N., Ortero C., Ortero S., Kim J. Equilibrium Effects of Food Labeling Policies. Proceedings of the 10th Annual Conference of the American Society of Health Economists.

[8] Reyes M., Smith Taillie L., Popkin B., Kanter R., Vandevijvere S., Corvalán C. (2020). Changes in the amount of nutrient of packaged foods and beverages after the initial implementation of the Chilean Law of Food Labelling and Advertising: A nonexperimental prospective study. PLoS Med..

[9] Shangguan S., Afshin A., Shulkin M., Ma W., Marsden D., Smith J., Saheb-Kashaf M., Shi P., Micha R., Imamura F. (2019). A Meta-Analysis of Food Labeling Effects on Consumer Diet Behaviors and Industry Practices. Am. J. Prev. Med..

[10] Paraje G., Colchero A., Wlasiuk J.M., Sota A.M., Popkin B.M. (2021). The effects of the Chilean food policy package on aggregate employment and real wages. Food Policy.

[11] Paraje G., Montes de Oca D., Wlasiuk J.M., Canales M., Popkin B.M. (2022). Front-of-Pack Labeling in Chile: Effects on Employment, Real Wages, and Firms’ Profits after Three Years of Its Implementation. Nutrients.

[12] Corvalán C., Correa T., Reyes M., Paraje G. (2021). Impacto de la ley Chilena de Etiquetado en el Sector Productivo Alimentario.

[13] Paraje G., Montes de Oca D., Corvalán C., Popkin B. (2022). Evolution of food and beverage prices after the front-of-package labeling regulations in Chile.

[14] Kanter R., Reyes M., Corvalán C. (2017). Photographic Methods for Measuring Packaged Food and Beverage Products in Supermarkets. Curr. Dev. Nutr..

[15] Van Ours J.C., Williams J. (2007). Cannabis prices and dynamics of cannabis use. J. Health Econ..

[16] Paternoster R., Brame R., Mazerolle P., Piquero A. (1998). Using the correct statistical test for the equality of regression coefficients. Criminology.

[17] Clogg C.C., Petkova E., Haritou A. (1995). Statistical Methods for Comparing Regression Coefficients Between Models. Am. J. Sociol..

[18] Carpentier F.R.D., Correa T., Reyes M., Taillie L.S. (2019). Evaluating the impact of Chile’s marketing regulation of unhealthy foods and beverages: Pre-school and adolescent children’s changes in exposure to food advertising on television. Public Health Nutr..

[19] Correa T., Reyes M., Taillie L.S., Corvalán C., Dillman Carpentier F.R. (2020). Food Advertising on Television Before and After a National Unhealthy Food Marketing Regulation in Chile, 2016–2017. Am. J. Public Health.

[20] Stoltze F.M., Barker J.O., Kanter R., Corvalán C., Reyes M., Taillie L.S., Carpentier F.R.D. (2017). Prevalence of child-directed and general audience marketing strategies on the front of beverage packaging: The case of Chile. Public Health Nutr..

[21] Ministerio de Salud de Chile (2017). Encuesta Nacional de Salud 2016–2017. Primeros Resultados.

[22] Caro J.C., Corvalán C., Reyes M., Silva A., Popkin B., Taillie L.S. (2018). Chile’s 2014 sugar-sweetened beverage tax and changes in prices and purchases of sugar-sweetened beverages: An observational study in an urban environment. PLoS Med..

[23] Hattersley L., Thiebaud A., Silver L., Mandeville K. (2020). Countering Common Argumentw against Taxes on Sugary Drinks.

[24] Fuchs A., Fernanda M., Icaza G. (2021). The Welfare and Distributional Effects of Taxing SSB to Reduce the Risks of Obesity in Ukraine.

[25] Fuchs A., Mandeville K., Alonso-Soria A. (2020). Health and Distributional Effects Taxing Sugar-Sweetened Beverages: The Case of Kazakhstan.

[26] Thow A.M., Downs S.M., Mayes C., Trevena H., Waqanivalu T., Cawley J. (2018). Fiscal Policy to Improve Diets and Prevent Noncommunicable Diseases: From Recommendations to Action. Bull. World Health Organ..

[27] Løvhaug A.L., Granheim S.I., Djojosoeparto S.K., Harrington J.M., Kamphuis C.B.M., Poelman M.P., Roos G., Sawyer A., Stronks K., Torheim L.E. (2022). The potential of food environment policies to reduce socioeconomic inequalities in diets and to improve healthy diets among lower socioeconomic groups: An umbrella review. BMC Public Health.

[28] Shekar M., Popkin B. (2020). Obesity: Health and Economic Consequences of an Impending Global Challenge.

[29] Jacobs A. In Sweeping War on Obesity, Chile Slays Tony the Tiger. *New York Times*, 7 February 2018. https://www.nytimes.com/2018/02/07/health/obesity-chile-sugar-regulations.html.

