# Supplementary material for: Socioeconomic Patterns in Budget Share Allocations of Regulated Foods and Beverages in Chile: A Longitudinal Analysis
Source: Nutrients. 2023 Jan 29;15(3):679. doi: 10.3390/nu15030679 (PMC9920097; doi:10.3390/nu15030679)
Supplement: Supplementary file 1 [file nutrients-15-00679-s001.zip › nutrients-2139801-supplementary.pdf]

SUPPLEMENTAL MATERIAL

Table S1: Complete results for the estimation of equation (2) for budget share.

	Regulated	Kcal	Saturated fat	Sodium	Sugar
Labeling and advertising law	-0.1255	-0.0484	-0.5211***	-0.0354	0.1015
	(0.3075)	(0.1943)	(0.1849)	(0.1390)	(0.2754)
Monthly trend	0.0000	0.0000***	0.0000	0.0000	0.0000***
	(0.0000)	(0.0000)	(0.0000)	(0.0000)	(0.0000)
Labeling and advertising law*Monthly trend	0.0000	0.0001	0.0007***	-0.0000	-0.0002
	(0.0005)	(0.0003)	(0.0003)	(0.0002)	(0.0004)
Socioeconomic level C2C3 (ref: ABC1)	-0.1461	-0.2011**	-0.0910	0.0350	-0.0924
	(0.1272)	(0.0803)	(0.0764)	(0.0575)	(0.1139)
Socioeconomic level DE (ref: ABC1)	-0.6229***	-0.1301	-0.0729	-0.1912***	-0.4527***
	(0.1448)	(0.0915)	(0.0870)	(0.0655)	(0.1297)
Socioeconomic level C2C3 (ref: ABC1) * Labeling and advertising law	0.6721**	0.5086***	0.3791**	0.1535	-0.2101
	(0.3045)	(0.1923)	(0.1830)	(0.1377)	(0.2726)
Socioeconomic level DE (ref: ABC1) * Labeling and advertising law	0.6936**	0.1570	0.2161	0.1445	0.0858
	(0.3412)	(0.2155)	(0.2051)	(0.1543)	(0.3056)
Socioeconomic level C2C3 (ref: ABC1) * Monthly trend	0.0002	0.0003***	0.0002	-0.0000	0.0001
	(0.0002)	(0.0001)	(0.0001)	(0.0001)	(0.0002)
Socioeconomic level DE (ref: ABC1) * Monthly trend	0.0009***	0.0002	0.0001	0.0003***	0.0006***
	(0.0002)	(0.0001)	(0.0001)	(0.0001)	(0.0002)

Socioeconomic level C2C3 (ref: ABC1) * Labeling and advertising law *Monthly trend	-0.0010**	-0.0008***	-0.0006**	-0.0002	0.0003
	(0.0004)	(0.0003)	(0.0003)	(0.0002)	(0.0004)
Socioeconomic level DE (ref: ABC1) * Labeling and advertising law *Monthly trend	-0.0010**	-0.0002	-0.0003	-0.0002	-0.0001
	(0.0005)	(0.0003)	(0.0003)	(0.0002)	(0.0004)
Couple with children <5 years (ref: couple without children)	0.3531	-0.0895	-0.0726	0.0254	0.2162
	(0.3213)	(0.2030)	(0.1932)	(0.1453)	(0.2877)
Couple with children 6-12 years (ref: couple without children)	0.0250	-0.1582*	0.0175	0.1159*	0.1230
	(0.1339)	(0.0846)	(0.0805)	(0.0605)	(0.1199)
Couple with children 13-17 years (ref: couple without children)	0.1830	0.1654	-0.0044	0.1146	0.1209
	(0.1607)	(0.1015)	(0.0966)	(0.0727)	(0.1439)
Couple with children 18-29 years (ref: couple without children)	0.0858	-0.0949	-0.0052	0.2961***	-0.0157
	(0.1490)	(0.0941)	(0.0895)	(0.0673)	(0.1334)
Mono-parental (ref: couple without children)	-0.2645	-0.1070	-0.0926	0.0756	-0.1622
	(0.1669)	(0.1054)	(0.1003)	(0.0755)	(0.1494)
Couple with children <5 years (ref: couple without children)* Labeling and advertising law	-0.5797	-0.5080	-0.1088	-0.2520	-0.1213
	(0.7240)	(0.4573)	(0.4352)	(0.3274)	(0.6483)
Couple with children 6-12 years (ref: couple without children)* Labeling and advertising law	0.0541	0.2646	0.1024	-0.1720	0.1249
	(0.3158)	(0.1995)	(0.1898)	(0.1428)	(0.2828)
Couple with children 13-17 years (ref: couple without children)* Labeling and advertising law	-0.3037	-0.3336	0.1214	-0.1750	-0.0453
	(0.3881)	(0.2452)	(0.2333)	(0.1755)	(0.3475)
Couple with children 18-29 years (ref: couple without	0.3996	0.4101**	0.0191	-0.3116**	0.6812**

children)* Labeling and advertising law	(0.3269)	(0.2065)	(0.1965)	(0.1478)	(0.2928)
Mono-parental (ref: couple without children)* Labeling and advertising law	0.4107	0.3927*	0.2013	-0.0185	0.3610
	(0.3725)	(0.2353)	(0.2239)	(0.1684)	(0.3336)
Couple with children <5 years (ref: couple without children)* Monthly trend	-0.0005	0.0001	0.0001	-0.0000	-0.0003
	(0.0005)	(0.0003)	(0.0003)	(0.0002)	(0.0004)
Couple with children 6-12 years (ref: couple without children)* Monthly trend	-0.0000	0.0002*	-0.0000	-0.0002*	-0.0002
	(0.0002)	(0.0001)	(0.0001)	(0.0001)	(0.0002)
Couple with children 13-17 years (ref: couple without children)* Monthly trend	-0.0003	-0.0003*	0.0000	-0.0002	-0.0002
	(0.0002)	(0.0002)	(0.0001)	(0.0001)	(0.0002)
Couple with children 18-29 years (ref: couple without children)* Monthly trend	-0.0001	0.0001	0.0000	-0.0004***	0.0000
	(0.0002)	(0.0001)	(0.0001)	(0.0001)	(0.0002)
Mono-parental (ref: couple without children)* Monthly trend	0.0004	0.0002	0.0001	-0.0001	0.0002
	(0.0003)	(0.0002)	(0.0002)	(0.0001)	(0.0002)
Couple with children <5 years (ref: couple without children)* Labeling and advertising law * Monthly trend	0.0008	0.0008	0.0001	0.0004	0.0001
	(0.0011)	(0.0007)	(0.0006)	(0.0005)	(0.0009)
Couple with children 6-12 years (ref: couple without children)* Labeling and advertising law * Monthly trend	-0.0001	-0.0004	-0.0001	0.0002	-0.0002
	(0.0005)	(0.0003)	(0.0003)	(0.0002)	(0.0004)
Couple with children 13-17 years (ref: couple without children)* Labeling and advertising law * Monthly trend	0.0004	0.0005	-0.0002	0.0003	0.0000
	(0.0006)	(0.0004)	(0.0003)	(0.0003)	(0.0005)
Couple with children 18-29 years (ref: couple without children)* Labeling and	-0.0006	-0.0006**	-0.0000	0.0005**	-0.0010**

advertising law * Monthly trend	(0.0005)	(0.0003)	(0.0003)	(0.0002)	(0.0004)
Mono-parental (ref: couple without children)* Labeling and advertising law * Monthly trend	-0.0006	-0.0006*	-0.0003	0.0000	-0.0005
	(0.0005)	(0.0003)	(0.0003)	(0.0002)	(0.0005)
Household size					
	-0.0016	0.0022***	-0.0002	-0.0019***	-0.0023**
	(0.0012)	(0.0007)	(0.0007)	(0.0005)	(0.0011)
Age head of HH					
	-0.0002	-0.0000	0.0001	0.0001	0.0000
	(0.0002)	(0.0001)	(0.0001)	(0.0001)	(0.0002)
% obese in HH					
	0.0018	0.0027	0.0044*	-0.0024	0.0001
	(0.0039)	(0.0025)	(0.0023)	(0.0018)	(0.0035)
Intercept					
	0.4165***	-0.2816***	0.0742	0.0958	0.6048***
	(0.1330)	(0.0840)	(0.0800)	(0.0602)	(0.1191)

*: $p < 0.1$, **: $p < 0.05$, ***: $p < 0.01$; Standard Deviation in parenthesis; Monthly dummies included in the regression, not included in the report.

Table S2: Marginal effect on the budget shares and trend before and after the intervention by type of labels

	Kcal.	Sat. fat.	Sodium	Sugar
Change in level postintervention	-0.0118***	-0.0306***	-0.0527***	-0.0717***
	(0.0016)	(0.0016)	(0.0012)	(0.0023)
Trend				
Preintervention	0.0009***	0.0002***	-0.0001***	-0.0003***
	(0.0000)	(0.0000)	(0.0000)	(0.0001)
Postintervention	0.0003***	0.0005***	-0.0001**	-0.0007***
	(0.0001)	(0.0001)	(0.0001)	(0.0001)
Difference between post and preintervention	-0.0006***	0.0002**	0.0000	-0.0004**
	(0.0001)	(0.0001)	(0.0001)	(0.0002)

*: p < 0.1, **: p < 0.05, ***: p < 0.01; Standard Deviation in parenthesis.

Table S3: Marginal effect on the budget shares and trend before and after the intervention by type of labels and SES level.

	Kcal.	Sat. fat.	Sodium	Sugar
Change in level ABC1				
	-0.0119***	-0.0363***	-0.0521***	-0.0710***
	(0.0037)	(0.0036)	(0.0027)	(0.0053)
Change in level C2C3				
	-0.0106***	-0.0306***	-0.0485***	-0.0739***
	(0.0022)	(0.0021)	(0.0016)	(0.0031)
Change in level DE				
	-0.0141***	-0.0267***	-0.0615***	-0.0678***
	(0.0029)	(0.0027)	(0.0021)	(0.0041)
Trend				
Difference between ABC1 and C2C3	-0.0014	-0.0057	-0.0036	0.0029
	(0.0043)	(0.0041)	(0.0031)	(0.0060)
Difference between C2C3 and DE	0.0035	-0.0040	0.0129***	-0.0060
	(0.0035)	(0.0033)	(0.0025)	(0.0050)
Difference between ABC1 and DE	0.0022	-0.0097**	0.0093**	-0.0032
	(0.0047)	(0.0044)	(0.0033)	(0.0066)
ABC1 preintervention	0.0007***	0.0001	-0.0002***	-0.0005***
	(0.0001)	(0.0001)	(0.0001)	(0.0001)
ABC1 postintervention	0.0005**	0.0007***	-0.0000	-0.0011***
	(0.0002)	(0.0002)	(0.0002)	(0.0003)
Difference between post and preintervention	-0.0001	0.0006**	0.0002	-0.0005
	(0.0002)	(0.0002)	(0.0002)	(0.0003)

C2C3 preintervention	0.0010***	0.0003***	-0.0002***	-0.0004***
	(0.0001)	(0.0001)	(0.0000)	(0.0001)
C2C3 postintervention	0.0001	0.0003***	-0.0003***	-0.0006***
	(0.0001)	(0.0001)	(0.0001)	(0.0002)
Difference between post and preintervention	-0.0009***	0.0001	-0.0001	-0.0002
	(0.0001)	(0.0001)	(0.0001)	(0.0002)
DE preintervention	0.0009***	0.0002**	0.0001**	0.0001
	(0.0001)	(0.0001)	(0.0001)	(0.0001)
DE postintervention	0.0006***	0.0005***	0.0001	-0.0005**
	(0.0002)	(0.0002)	(0.0001)	(0.0003)
Difference between post and preintervention	-0.00037*	0.00031*	-0.00007	-0.00063**
	(0.0002)	(0.0002)	(0.0001)	(0.0003)

*: p < 0.1, **: p < 0.05, ***: p < 0.01; Standard Deviation in parenthesis.

Table S4: Average monthly volume purchased of high in- products by period and by SES level.

	Solid Food (gr)						Beverages (ml)	
**Total sample**								
Volume	No Label	Any Label	Kcal	Sat Fat	Sodium	Sugar	No Label	Sugar
Pre-intervention average	15586.19	5287.09	2601.606	2375.836	1902.949	2241.96	35580.62	14584.35
	(10193.81)	(3758.983)	(2052.005)	(1873.169)	(1828.975)	(2138.816)	(29211.65)	(16230.36)
Post-intervention average	16514.54	4093.697	2183.178	1921.951	531.5498	1730.18	35255.85	9797.91
	(11288.04)	(3154.498)	(1848.846)	(1685.661)	(799.4643)	(1763.157)	(28434.31)	(13128.05)
Difference of coefficients	928.3546	- 1193.393	-418.4288	-453.88	- 1371.39	-511.774	324.7727	- 4786.443
Standard errors	72.16	24.11	13.45	12.27	10.40	13.65	198.83	103.1869
**ABC1**								
	Solid Food (gr)						Beverages (ml)	
	No Label	Any Label	Kcal	Sat Fat	Sodium	Sugar	No Label	Sugar
Pre-intervention average	15195.39	6064.98	3138.58	2702.46	1827.72	2765.65	46161.85	13055.16
	(9651.31)	(3979.62)	(2234.03)	(1999.42)	(1790.83)	(2339.98)	(34707.69)	(14944.37)
Post-intervention average	15287.19	4459.41	2526.05	2002.18	428.99	1903.45	43937.28	6369.79
	(9941.27)	(3143.11)	(1900.89)	(1623.09)	(686.63)	(1842.92)	(32492.83)	(8605.58)
Difference of coefficients	91.80	-1605.57	-612.54	-700.28	-1398.73	-862.20	2224.563	-6685.38
Standard errors	171.37	64.84	37.17	32.82	26.02	38.10	599.0184	228.20
**C2C3**								
	Solid Food (gr)						Beverages (ml)	
	No Label	Any Label	Kcal	Sat Fat	Sodium	Sugar	No Label	Sugar
Pre-intervention average	15480.42	5366.30	2688.86	2413.83	1856.46	2323.50	35640.62	14670.88
	(10241.49)	(3790.65)	(2105.41)	(1899.68)	(1778.86)	(2207.72)	(28816.63)	(16407.50)
Post-intervention average	16525.43	4160.41	2263.91	1930.71	504.47	1803.43	35770.36	9844.35
	(11313.80)	(3226.96)	(1900.50)	(1705.71)	(773.43)	(1832.20)	(28724.57)	(12899.76)
Difference of coefficients	1045.01	-1205.88	-424.95	-483.12	-1351.99	-520.07	129.734	-4826.53

Standard errors	100.11	33.79	19.11	17.21	14.02	19.54	273.3588	143.27
	DE							
	Solid Food (gr)						Beverages (ml)	
	No Label	Any Label	Kcal	Sat Fat	Sodium	Sugar	No Label	Sugar
Pre-intervention average	15945.56	4789.26	2204.00	2158.96	2015.41	1860.08	30362.9	15163.29
	(10352.07)	(3519.56)	(1780.20)	(1735.90)	(1921.25)	(1835.28)	(25309.88)	(16472.77)
Post-intervention average	17051.07	3824.25	1902.08	1872.05	620.23	1537.32	30480.31	11268.71
	(11766.78)	(3020.06)	(1698.58)	(1680.27)	(874.80)	(1591.09)	(24737.66)	(14780.92)
Difference of coefficients	1105.50	-3020.06	-301.92	-286.91	-1395.18	-322.77	117.4158	-3894.58
Standard errors	129.74	39.70	20.80	20.39	1395.18	20.77	302.8082	188.79

Standard Deviation in parenthesis

Table S5: Marginal effect of the intervention on the volume purchased of labelled products and trends before and after the intervention

Any label	Change in level postintervention	Trends		
		Preintervention trend	Postintervention trend	Difference between post and preintervention trends
Solid foods	-1458.0937*** (59.0958)	15.4319*** (1.7356)	-4.6314 (3.4659)	-20.0633*** (3.8035)
Beverages	-3627.9186*** (204.3940)	-49.8319*** (5.9934)	-93.0930*** (11.9821)	-43.2611*** (13.1462)

*: $p < 0.1$, **: $p < 0.05$, ***: $p < 0.01$; Standard errors in parenthesis.

Table S6: Marginal effect of the intervention on the volume purchased and trend before and after the intervention by label and by SES level.

	Solid Foods, any label			Beverages, high in sugar		
	ABC1	C2C3	DE	ABC1	C2C3	DE
Change in level any label	-1878.5539***	-1312.0308***	-1475.2935***	-4543.9111***	-3720.7061***	-2846.3251***
	(134.5928)	(78.1546)	(103.7442)	(465.7299)	(270.3313)	(358.7327)
Any label: Difference between	ABC1 and C2C3	C2C3 and DE	ABC1 and DE	ABC1 and C2C3	C2C3 and DE	ABC1 and DE
	-566.523***	163.2627	-403.2604**	-823.2049	-874.3811**	-1697.586***
	(152.7230)	(126.0710)	(167.3927)	(528.4204)	(436.0102)	(579.1049)
	ABC1	C2C3	DE	ABC1	C2C3	DE
Any label: preintervention trend	1.1112	14.8238***	26.4226***	-103.3621***	-40.5660***	-32.4089***
	(3.7812)	(2.2493)	(3.2056)	(13.0396)	(7.7704)	(11.0729)
Any label: postintervention trend	4.8083	-12.6999***	4.9185	-70.9592**	-100.8513***	-91.8253***
	(7.9740)	(4.6188)	(6.3470)	(27.5666)	(15.9688)	(21.9482)
Difference between post and preintervention	3.697	-27.5237***	-21.5041***	32.403	-60.2853***	-59.4164**
	(8.8002)	(5.0838)	(7.0870)	(30.4061)	(17.5723)	(24.5048)

*: $p < 0.1$, **: $p < 0.05$, ***: $p < 0.01$; Standard errors in parenthesis.

Table S7: Marginal effect of the intervention on the volume purchased and trend before and after the intervention by "life cycle".

	Solid foods, any label					
	HH without children	Couple with children <5 years	Couple with children 6-12 years	Couple with children 13-17 years	Couple with children 18-29 years	Mono-parental with children
Change in level	-1274.1568***	-459.3067	-1685.9807***	-1733.3565***	-1549.1665***	-1399.8738***
	(94.0589)	(410.8805)	(128.3386)	(180.6702)	(119.9210)	(147.7415)
Preintervention trend	11.1419***	2.9060	23.1883***	10.0393**	8.5006**	28.6925***
	(3.0389)	(10.6063)	(3.4560)	(4.6209)	(4.0785)	(4.8108)
Postintervention trend	4.5316	-15.3250	-14.2463*	-6.0599	2.1472	-15.8842*
	(5.9460)	(20.2580)	(7.4438)	(10.1183)	(7.6122)	(9.2053)
Difference between pre and post intervention	-6.6103	-18.2310	-37.4346***	-16.0992	-6.3534	-44.5767***
	(6.6558)	(23.1009)	(8.1391)	(11.0719)	(8.6323)	(10.4145)
	Beverages, high in sugar					
	HH without children	Couple with children <5 years	Couple with children 6-12 years	Couple with children 13-17 years	Couple with children 18-29 years	Mono-parental with children
Change in level	-3083.9562***	-2803.1293**	-4296.6093***	-4920.9547***	-3254.3803***	-3059.2614***
	(326.1039)	(0.0014)	(443.0073)	(624.5325)	(414.3183)	(510.3417)
Preintervention trend	-24.9776**	-125.5533***	-36.5439***	-98.2955***	-98.2283***	15.0085
	(10.5105)	(36.6577)	(11.9284)	(15.9519)	(14.0830)	(16.5783)
Postintervention trend	-61.4838***	-226.5621***	-125.9116***	-77.7226**	-94.8549***	-82.5319***

	(20.6030)	(70.0188)	(25.7093)	(34.9756)	(26.2984)	(31.7791)
Difference between pre and post intervention	-36.5062	-101.0088	-89.3678***	20.573	3.3735	-97.5404**
	(23.0533)	(79.7963)	(28.1062)	(38.2595)	(29.8174)	(35.9432)

*: p < 0.1, **: p < 0.05, ***: p < 0.01; Standard errors in parenthesis.

Table S8: Marginal effect of the intervention on the monthly volume purchased in labelled products and trends before and after the intervention by type of label

	Kcal.	Sat. fat.	Sodium	Sugar	Beverages
Change in level postintervention	-722.7257***	-692.3386***	-1327.3114***	-583.1729***	-3627.9186***
	(34.1988)	(31.5377)	(27.2420)	(36.1169)	(204.3940)
Trend					
Preintervention	12.9571***	4.4836***	0.7830	4.9406***	-49.8319***
	(1.0044)	(0.9262)	(0.8001)	(1.0607)	(5.9934)
Postintervention	5.4411***	5.2140***	-4.2992***	-6.2816***	-93.0930***
	(2.0057)	(1.8497)	(1.5977)	(2.1182)	(11.9821)
Difference between post and preintervention	-7.5160***	0.7305	-5.0822***	-11.2223***	-43.2611***
	(2.2011)	(2.0298)	(1.7533)	(2.3245)	(13.1462)

*: p < 0.1, **: p < 0.05, ***: p < 0.01; Standard Deviation in parenthesis.

Table S9: Marginal effect of the intervention on the volume purchased and trend before and after the intervention by type of label and by SES level.

	Kcal.	Sat. fat.	Sodium	Sugar	Beverages
Change in level ABC1					
	-900.0242***	-1018.7511***	-1389.4491***	-748.0578***	-4543.9111***
	(77.8890)	(71.8283)	(62.0447)	(82.2575)	(465.7299)
Change in level C2C3					
	-685.2494***	-639.0710***	-1226.1818***	-578.5882***	-3720.7061***
	(45.2281)	(41.7089)	(36.0278)	(47.7648)	(270.3313)
Change in level DE					
	-681.9516***	-585.9496***	-1488.2697***	-484.9735***	-2846.3251***
	(60.0369)	(55.3653)	(47.8241)	(63.4041)	(358.7327)
Difference between ABC1 and C2C3	-214.7748**	-379.6800***	-163.2673**	-169.4696*	-823.2049
	(88.3809)	(81.5039)	(70.4024)	(93.3379)	(528.4204)
Difference between C2C3 and DE	-3.2978	-53.1214	262.0879***	-93.6147	-874.3811**
	(72.9574)	(67.2805)	(58.1163)	(77.0493)	(436.0102)
Difference between DE and ABC1	218.0726**	432.8015***	-98.8206	263.0843**	-1697.586***
	(96.8703)	(89.3327)	(77.1649)	(102.3035)	(579.1049)
Trend					
ABC1 preintervention	4.7421**	-1.0288	-2.9258*	-3.3704	-103.3621***
	(2.1882)	(2.0179)	(1.7431)	(2.3109)	(13.0396)
ABC1 postintervention	9.3194**	14.3131***	-3.8163	-10.1919**	-70.9592**
	(4.6146)	(4.2555)	(3.6759)	(4.8734)	(27.5666)
Difference between post and preintervention	4.5773	15.3419***	-0.8904	-6.8215	32.403
	(5.0927)	(4.6964)	(4.0567)	(5.3783)	(30.4061)

C2C3 preintervention	14.7361***	5.9009***	-1.0157	4.8142***	-40.5660***
	(1.3017)	(1.2004)	(1.0369)	(1.3747)	(7.7704)
C2C3 postintervention	2.0688	0.2351	-6.9557***	-6.8931**	-100.8513***
	(2.6729)	(2.4649)	(2.1292)	(2.8228)	(15.9688)
Difference between post and preintervention	-12.6673***	-5.6657**	-5.9400**	-11.7074***	-60.2853***
	(2.9420)	(2.7131)	(2.3436)	(3.1070)	(17.5723)
DE preintervention	14.8856***	5.3178***	7.0049***	10.8533***	-32.4089***
	(1.8551)	(1.7107)	(1.4777)	(1.9591)	(11.0729)
DE postintervention	9.4732***	9.1191***	0.4388	-2.7493	-91.8253***
	(3.6730)	(3.3872)	(2.9258)	(3.8790)	(21.9482)
Difference between post and preintervention	-5.4124	3.8013	-6.5662**	-13.6026***	-59.4164**
	(4.1013)	(3.7821)	(3.2670)	(4.3313)	(24.5048)

*: p < 0.1, **: p < 0.05, ***: p < 0.01; Standard Deviation in parenthesis.

Table S10: Marginal effect of the intervention on the volume purchased and trend before and after the intervention by label and by life cycle.

	Kcal.	Sat. fat.	Sodium	Sugar	Beverages
Change in level HH without children	-652.7823***	-609.8034***	-974.0869***	-608.0621***	-3083.9562***
	(54.4320)	(50.1966)	(43.3594)	(57.4849)	(326.1039)
Change in level couple with children <5 years	-647.6981***	-466.6363**	-1469.5928***	130.8223	-2803.1293**
	(237.7770)	(219.2752)	(189.4082)	(251.1131)	(0.0014)
Change in level couple with children 6-12 years	-716.2471***	-734.9001***	-1529.8020***	-712.7862***	-4296.6093***
	(74.2696)	(68.4906)	(59.1617)	(78.4352)	(443.0073)
Change in level couple with children 13-17 years	-1005.5862***	-855.0235***	-1564.7688***	-591.5525***	-4920.9547***
	(104.5540)	(96.4185)	(83.2855)	(110.4181)	(624.5325)
Change in level couple with children 18-29 years	-770.6953***	-772.5395***	-1389.1790***	-585.6402***	-3254.3803***
	(69.3984)	(63.9984)	(55.2813)	(73.2907)	(414.3183)
Change in level monoparental with children	-562.1074***	-604.6589***	-1367.0579***	-530.1831***	-3059.2614***
	(85.4982)	(78.8454)	(68.1061)	(90.2935)	(510.3417)
HH without children preintervention	9.2796***	4.3449***	3.3870**	1.7918	-24.9776**
	(1.7586)	(1.6218)	(1.4009)	(1.8573)	(10.5105)
HH without children postintervention	7.6620**	8.4281***	-1.5959	2.2712	-61.4838***
	(3.4410)	(3.1732)	(2.7410)	(3.6340)	(20.6030)
Difference between post and preintervention	-1.6176	4.0833	-4.9829	0.4795	-36.5062
	(3.8517)	(3.5520)	(3.0682)	(4.0678)	(23.0533)

Couple with children <5 years preintervention	9.8653	2.5164	1.5531	-2.3674	-125.5533***
	(6.1379)	(5.6603)	(4.8893)	(6.4821)	(36.6577)
Couple with children <5 years postintervention	15.4235	-0.8532	-6.7422	-17.8309	-226.5621***
	(11.7233)	(10.8111)	(9.3385)	(12.3808)	(70.0188)
Difference between post and preintervention	5.5582	-3.3696	-8.2953	-15.4635	-101.0088
	(13.3685)	(12.3283)	(10.6491)	(14.1183)	(79.7963)
Couple with children 6-12 years preintervention	18.5610***	8.2005***	2.7538*	6.1570***	-36.5439***
	(2.0000)	(1.8444)	(1.5932)	(2.1122)	(11.9284)
Couple with children 6-12 years postintervention	-1.9251	-0.1737	-7.5406**	-12.0839***	-125.9116***
	(4.3077)	(3.9725)	(3.4314)	(4.5493)	(25.7093)
Difference between post and preintervention	-20.4861***	-8.3741*	-10.2944**	-18.2408***	-89.3678***
	(4.7101)	(4.3436)	(3.7520)	(4.9743)	(28.1062)
Couple with children 13-17 years preintervention	9.3458***	0.2516	-3.9330*	4.9978*	-98.2955***
	(2.6741)	(2.4660)	(2.1301)	(2.8241)	(15.9519)
Couple with children 13-17 years postintervention	14.4834**	2.5484	-3.0600	-7.0520	-77.7226**
	(5.8554)	(5.3998)	(4.6643)	(6.1839)	(34.9756)
Difference between post and preintervention	5.1376	2.2968	0.873	-12.0498*	20.573
	(6.4073)	(5.9087)	(5.1039)	(6.7667)	(38.2595)
Couple with children 18-29 years preintervention	10.9470***	0.3939	-7.1408***	5.6759**	-98.2283***
	(2.3602)	(2.1766)	(1.8801)	(2.4926)	(14.0830)
Couple with children 18-29 years postintervention	7.2965*	8.6450**	-2.1250	-5.7286	-94.8549***
	(4.4052)	(4.0624)	(3.5091)	(4.6523)	(26.2984)

Difference between post and preintervention	-3.6505<br>(4.9955)	8.2512*<br>(4.6068)	5.0158<br>(3.9793)	-11.4045**<br>(5.2757)	3.3735<br>(29.8174)
Monoparental with children preintervention	17.3482***<br>(2.7840)	7.9192***<br>(2.5674)	6.2661***<br>(2.2177)	11.2645***<br>(2.9401)	15.0085<br>(16.5783)
Monoparental with children postintervention	-1.8913<br>(5.3271)	5.0852<br>(4.9126)	-8.8152**<br>(4.2435)	-13.2865**<br>(5.6259)	-82.5319***<br>(31.7791)
Difference between post and preintervention	-19.2396***<br>(6.0269)	-2.8340<br>(5.5579)	-15.0813***<br>(4.8009)	-24.551***<br>(6.3649)	-97.5404**<br>(35.9432)

*: p < 0.1, **: p < 0.05, ***: p < 0.01; Standard Deviation in parenthesis.
